# Supplementary material for: Current and future goals are represented in opposite patterns in object-selective cortex
Source: eLife. 2018 Nov 6;7:e38677. doi: 10.7554/eLife.38677 (PMC6279347; doi:10.7554/eLife.38677)
Supplement: Transparent reporting form [file elife-38677-transrepform.docx]

***eLife’s* transparent reporting form**

We encourage authors to provide detailed information *within their submission* to facilitate the interpretation and replication of experiments. Authors can upload supporting documentation to indicate the use of appropriate reporting guidelines for health-related research (see [EQUATOR Network](http://www.equator-network.org/%20)), life science research (see the [BioSharing Information Resource](https://biosharing.org/)), or the [ARRIVE guidelines](http://www.plosbiology.org/article/info:doi/10.1371/journal.pbio.1000412) for reporting work involving animal research. Where applicable, authors should refer to any relevant reporting standards documents in this form.

If you have any questions, please consult our Journal Policies and/or contact us: [editorial@elifesciences.org](mailto:editorial@elifesciences.org).

**Sample-size estimation**

- You should state whether an appropriate sample size was computed when the study was being designed
- You should state the statistical method of sample size computation and any required assumptions
- If no explicit power analysis was used, you should describe how you decided what sample (replicate) size (number) to use

Please outline where this information can be found within the submission (e.g., sections or figure legends), or explain why this information doesn’t apply to your submission:

We decided on the sample size of N= 24 (Experiment 1) and N = 25 (Experiment 2) based on previous studies using similar methods (Albers et al. 2013 Curr. Bio Lee et al. 2013 NN, van Loon et al. Cerebral Cortex)

**Replicates**

- You should report how often each experiment was performed
- You should include a definition of biological versus technical replication
- The data obtained should be provided and sufficient information should be provided to indicate the number of independent biological and/or technical replicates
- If you encountered any outliers, you should describe how these were handled
- Criteria for exclusion/inclusion of data should be clearly stated
- High-throughput sequence data should be uploaded before submission, with a private link for reviewers provided (these are available from both GEO and ArrayExpress)

Please outline where this information can be found within the submission (e.g., sections or figure legends), or explain why this information doesn’t apply to your submission:

-Each experiment was performed one time

-We did not encounter any outliers, all participants were included in the fMRI analysis and behavioral analysis. In Experiment 1, the object-selective cortex mapper data of 2 participants was not usable therefore we used anatomical masks instead. We report this in our manuscript in the method section: ‘*Regions of Interest: object-selective cortex mapper (pFs)* ‘

-To our knowledge biological or technical replication does not apply to our study

**Statistical reporting**

- Statistical analysis methods should be described and justified
- Raw data should be presented in figures whenever informative to do so (typically when N per group is less than 10)
- For each experiment, you should identify the statistical tests used, exact values of N, definitions of center, methods of multiple test correction, and dispersion and precision measures (e.g., mean, median, SD, SEM, confidence intervals; and, for the major substantive results, a measure of effect size (e.g., Pearson's r, Cohen's d)
- Report exact p-values wherever possible alongside the summary statistics and 95% confidence intervals. These should be reported for all key questions and not only when the p-value is less than 0.05.

Please outline where this information can be found within the submission (e.g., sections or figure legends), or explain why this information doesn’t apply to your submission:

This can be found throughout the manuscript where the results areas discussed as well as in the method section. We report the N, mean, within-subject SEM and the exact p-values and Cohen’s d for the major substantive results.

(For large datasets, or papers with a very large number of statistical tests, you may upload a single table file with tests, Ns, etc., with reference to sections in the manuscript.)

**Group allocation**

- Indicate how samples were allocated into experimental groups (in the case of clinical studies, please specify allocation to treatment method); if randomization was used, please also state if restricted randomization was applied
- Indicate if masking was used during group allocation, data collection and/or data analysis

Please outline where this information can be found within the submission (e.g., sections or figure legends), or explain why this information doesn’t apply to your submission:

This does not apply to our submission, since we did not allocate participants into experimental groups

**Additional data files (“source data”)**

- We encourage you to upload relevant additional data files, such as numerical data that are represented as a graph in a figure, or as a summary table
- Where provided, these should be in the most useful format, and they can be uploaded as “Source data” files linked to a main figure or table
- Include model definition files including the full list of parameters used
- Include code used for data analysis (e.g., R, MatLab)
- Avoid stating that data files are “available upon request”

Please indicate the figures or tables for which source data files have been provided:

**EXPERIMENT 1:**

-**Table 1 reaction time and accuracy Experiment 1:** Table 1–source data 1

- **Figure 2,** **Classification Performance**: Figure2_WithinCrossRelevanceDecoding.xlsx, sheet withindecoding, Figure2_WithinCrossRelevanceDecodin.xlsx, sheet crossdecoding. Source code: Figure2-source data 1.zip

**- Figure 2 –figure supplement 1, Mean BOLD:** FigureS1_MeanBold.xlsx, sheet MeanBOLD. Source code: Figure2- source data 2.zip

**- Figure 2 –figure supplement 2, Cross-temporal generalization of object category decoding** : FigureS2_CrossTemporalGeneralization.xlsx, sheet CrossTemporalGeneralization, Source code: Figure2-source data 3.zip

**- Figure 3ABC, RDM & Histogram, =>** Figure3_RDM.xlsx, sheet RDM & Histogram

Source code: Figure3-source data 1.zip

The MVPA data (for all participants, voxel x activation x condition) are stored in an ‘NNStructs’ but these files are too large to upload via the submission system, these will be provided via the open science framework:

*Van Loon, Anouk M. 2018. “Current and Future Goals Are Represented in Opposite Patterns in Object-Selective Cortex.” Open Science Framework. May 31. osf.io/hcp47.*

In the source code scripts there are references to these structures

Experiment 1: matlab structure: MVPA_pFs.zip => 'NNLOCOmb.mat' => pFs

**EXPERIMENT 2:**

-**Table 2 reaction time and accuracy Experiment 2:** Table2–source data 1

- **Figure 4** **Classification Performance**: Figure 4 source data 1 (contains matlab matrix with average per participant contains matlab scripts to reproduce Figure 4 and SPSS file to preform statistics).

**- Figure 4, figure supplement 1, Mean BOLD:** Figure 4- source data 2 (contains matlab matrix with average BOLD per participant and matlab scripts to reproduce Figure 4 and SPSS files to preform statistics).

**- Figure 5 RDM, scaling plots, histograms:** Figure 5 source data 1 (contains matlab matrix with average RDMs per participant and matlab scripts to reproduce Figure 5 and SPSS file to preform statistics).

The MVPA data (for all participants, voxel x activation x condition) are stored in an ‘NNStructs’ but these files are too large to upload via the submission system, these will be provided via the open science framework:

*Van Loon, Anouk M. 2018. “Current and Future Goals Are Represented in Opposite Patterns in Object-Selective Cortex.” Open Science Framework. May 31. osf.io/hcp47.*

In the source code scripts there are references to these structures

Experiment 2: matlab structure: MVPA_pFsExperiment2.zip => ' NNALLTR.mat' => pFs
